# Supplementary material for: Tubes and bubbles topological confinement of YouTube recommendations
Source: PLoS One. 2020 Apr 21;15(4):e0231703. doi: 10.1371/journal.pone.0231703 (PMC7173795; doi:10.1371/journal.pone.0231703)
Supplement: Attachment — Submitted filename: cover-letter-revision-including-response.pdf [file pone.0231703.s001.pdf]

C. Roth, A. Mazières and T. Menezes
CNRS
Centre Marc Bloch (CNRS/HU)
Computational Social Science team
Friedrichstrasse 191
10117 Berlin
Germany

March 17, 2020

Dear Editor,
Dear Reviewer,

We thank you for your consideration of our manuscript and are very pleased to have carried out the requested revision. We also fully agree with all the remarks of Reviewer 1, whom we wish to thank for his report on our work.

This revision attemps to improve the manuscript by taking into account all the issues that have been raised. In particular, we changed the wording related to the dynamics of recommendation graphs — it is true that confirming the contraction of these graphs over time *because* of user interaction would require a different protocol, where we would collect and study recommendation graphs in a longitudinal manner. In this respect, we amended our remarks to reflect the fact that we simply observed correlations between views and graph size and density, and that we cannot directly conclude on graph contraction, especially on pages 7, 8, 11 (previously 10), and 13.

We also added a clarification sentence regarding what we mean with "audience effect" on page 10 (previously 9), and toned down several claims identified by Reviewer 1. We further inserted two citations to two additional studies published in 2020 which feature the computation of diversity or confinement through random walks.

More broadly, we also took into account the general remark pertaining to our goals and to the framing of our work within the literature — we added sentences to this effect in the abstract, in the introduction, and in the conclusion. We hope that this further clarifies our positioning within the state of the art on the effects of algorithmic recommendation.

We finally added a precision regarding the respect of YouTube Terms of Service for the data collection operations. We mentioned the availability of our data and scripts in publicly-available repositories.

We emphasized all these changes by putting the text in blue where needed (for the sake of clarity, we however did not emphasize the very minor typo and form corrections).

We hope to have addressed all matters of concern regarding this manuscript in order to make it fit for publication. We nevertheless remain at your disposal to carry out further improvements.

Sincerely,

C. Roth, A. Mazières and T. Menezes
